# Porosome in Cystic Fibrosis

**DOI:** 10.15190/d.2014.16

**Published:** 2014-09-28

**Authors:** Bhanu P. Jena

**Affiliations:** Wayne State University School of Medicine, Department of Physiology, Detroit, MI, USA

**Keywords:** porosome, membrane fusion, cell secretion, exocytosis, membrane traffic

## Abstract

Macromolecular structures embedded in the cell plasma membrane called ‘porosomes’, are involved in the regulated fractional release of intravesicular contents from cells during secretion. Porosomes range in size from 15 nm in neurons and astrocytes to 100-180 nm in the exocrine pancreas and neuroendocrine cells. Porosomes have been isolated from a number of cells, and their morphology, composition, and functional reconstitution well documented. The 3D contour map of the assembly of proteins within the porosome complex, and its native X-ray solution structure at sub-nm resolution has also advanced. This understanding now provides a platform to address diseases that may result from secretory defects. Water and ion binding to mucin impart hydration, critical for regulating viscosity of the mucus in the airways epithelia. Appropriate viscosity is required for the movement of mucus by the underlying cilia. Hence secretion of more viscous mucus prevents its proper transport, resulting in chronic and fatal airways disease such as cystic fibrosis (CF). CF is caused by the malfunction of CF transmembrane conductance regulator (CFTR), a chloride channel transporter, resulting in viscous mucus in the airways. Studies in mice lacking functional CFTR secrete highly viscous mucous that adhered to the epithelium. Since CFTR is known to interact with the t-SNARE protein syntaxin-1A, and with the chloride channel CLC-3, which are also components of the porosome complex, the interactions between CFTR and the porosome complex in the mucin-secreting human airway epithelial cell line Calu-3 was hypothesized and tested. Results from the study demonstrate the presence of approximately 100 nm in size porosome complex composed of 34 proteins at the cell plasma membrane in Calu-3 cells, and the association of CFTR with the complex. In comparison, the nuclear pore complex measures 120 nm and is comprised of over 500 protein molecules. The involvement of CFTR in porosome-mediated mucin secretion is hypothesized, and is currently being tested.

## SUMMARY

1. Introduction
2. Porosome in Calu-3 cell
3. Ongoing studies

## 1. Introduction

**Figure 1:**
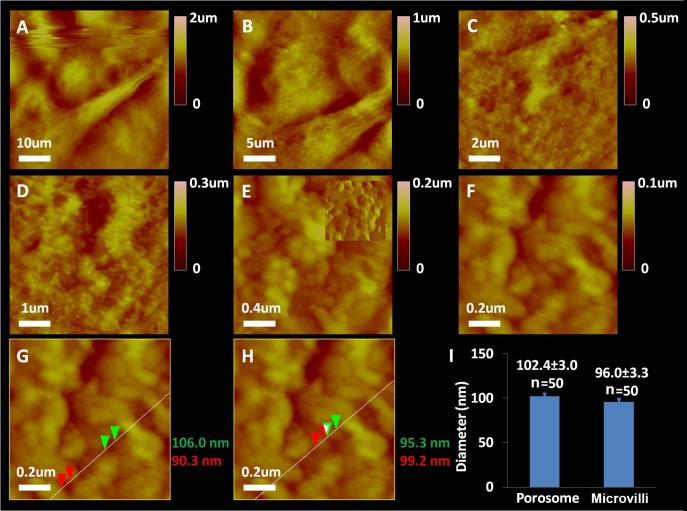
Atomic force microscopy (AFM) micrographs demonstrate the presence of microvilli and interspersed mucin-secreting porosomes at plasma membrane in Calu-3 cells^11^ Microvilli measuring on average 96 nm in thickness (mean ± SEM; 96 ± 3.3, n=50) are densely packed at the cell plasma membrane exposed to the medium, and is demonstrated both in low (A-C) and high (D-H) resolution AFM images. Interspersed among the microvilli are the 102 nm in diameter porosome openings (mean ± SEM; 102 ± 3, n=50) shown in figure G (red and green arrowheads). Similarly, the microvilli shown in figure H (red and green arrowheads) demonstrate some that appear coiled around each other, possibly as a consequence of secreted mucus^11^. ©Bhanu Jena.

**Figure 2:**
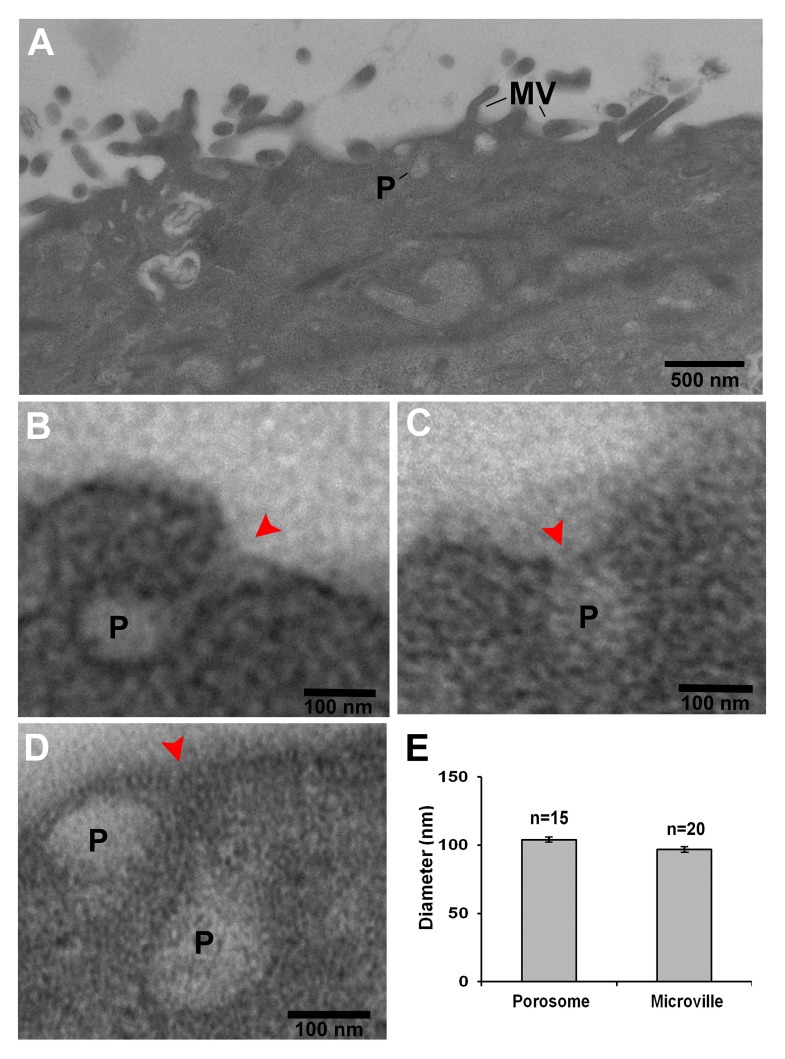
Representative electron micrographs of Calu-3 cells in culture demonstrating the presence of microvilli (MV) and porosomes (P) at the cell plasma membrane^11^ (A) Calu-3 cells demonstrate the presence of dense microvilli and scattered porosomes at the cell plasma membrane. (B-D) Note the flask-shaped porosomes measuring nearly 100 nm in diameter (E) and from 200-300 nm in depth, with openings to the cell surface (red arrowhead). Mucus (C), is found at the opening of the porosome to the cell exterior. Of the two porosomes shown in (D), the one to the center appears to be sectioned right through the center of the organelle, where as the porosome to the left, has been sectioned at its base. (E) Similar to the AFM images in Figure 1, the microvilli measure on average 92 nm in diameter^11^. ©Bhanu Jena.

**Figure 3:**
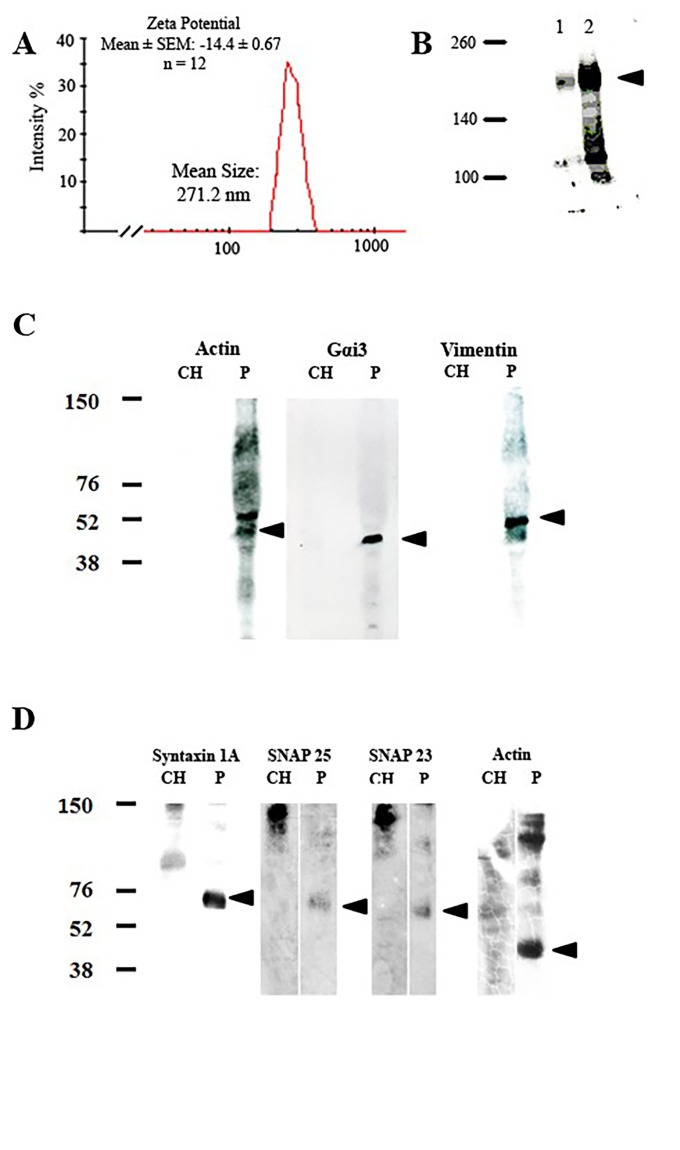
Photon correlation spectroscopy (PCS) demonstrate the immunoisolated porosome complex from Calu-3 cells to measure on average 271 nm (trimmers), and both immuno-precipitation and immunoblot analysis demonstrate the interaction of CFTR with the porosome complex in the cell^11^. (A) PCS on isolated porosomes from Calu-3 cells demonstrate an average size of 271.2 nm. (B) Immunoblot analysis using CFTR-specific antibody of CFTR-expressing HEK cell proteins resolved using SDS-PAGE, followed by electrotransfer to nitrocellulose membrane, demonstrates the presence of a 180 kD band representing CFTR (lane 1, positive control). SDS-PAGE resolved immunoisolated porosome complexes also demonstrate immunopositive for CFTR (lane 2). (C) Immunoblot analysis of the total Calu-3 cell homogenate (CH) and isolated porosome complex (P), demonstrate the presence of porosome proteins actin, Gai3, and vimentin. Note the enriched presence of the proteins in the porosome complex. (D) Similarly, immunoisolated CFTR complex using the CFTR-specific antibody, results in the pull-down of porosome associated proteins such as Syntaxin-1A (present as 70 kDa t-/v-SNARE complex), SNAP-25 (present as 70 kDa t-/v-SNARE complex), SNAP-23, present as 68 kDa t-/v-SNARE complex, and actin^11^. ©Bhanu Jena.

**Figure 4:**
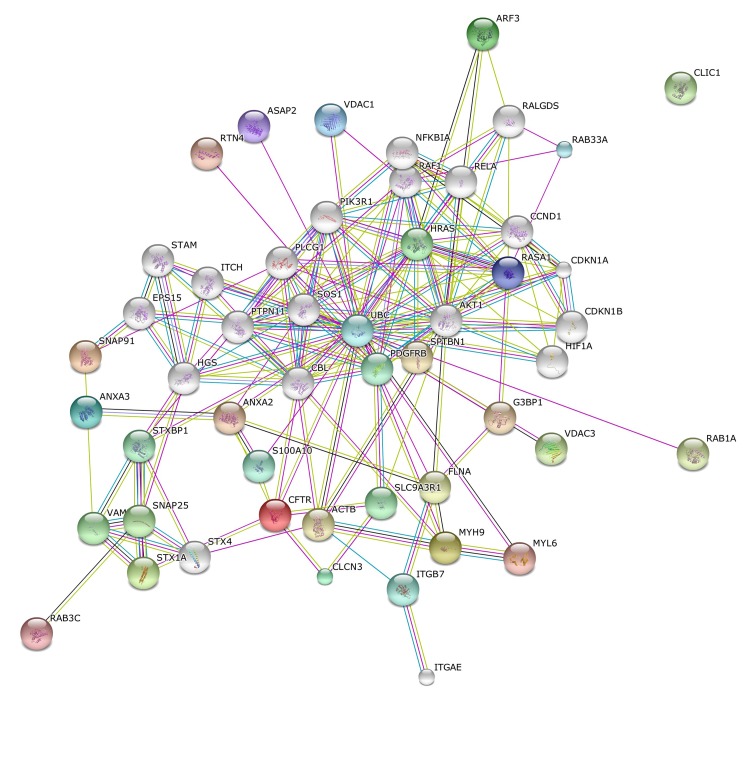
Schematic drawing depicting the evidence view of predicted interactions between identified proteins within the mucous-secreting Calu-3 porosome proteome and other regulatory proteins These interactions are generated from inputs of the identified proteins in the Calu-3 porosome, using STRING 9.1^7,11^. STRING 9.1 is a database of known functional and predicted protein-protein interactions. The interactions include direct (physical) and indirect (functional) associations derived from genomic, high-throughput, conserved co-expression, and earlier knowledge. Note the two clusters of protein-protein interactions identified in the porosome complex. The one cluster to the top, and most likely present at the apical end of the porosome cup are cytoskeletal structure and signalling proteins. The bottom cluster represents proteins that are primarily involved in membrane fusion including SNARE proteins and CFTR, and therefore their location would be at the base of the porosome complex facing the cytosol. The confidence of the predicted functional interactions shown are >99%. ©Bhanu Jena.

**Figure 5:**
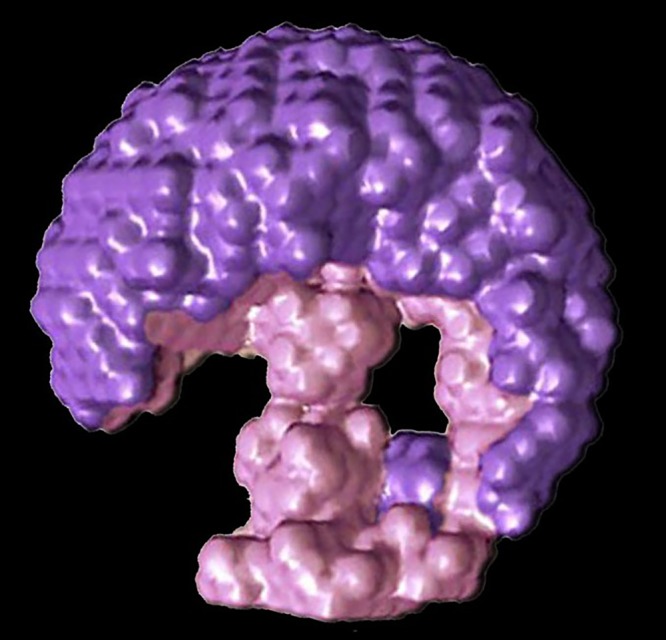
Small angle X-ray solution scattering structure of a native 35 nm synaptic vesicle (violet) docked with a 15 nm neuronal porosome complex (pink) at the presynaptic membrane Note the prominent central plug of the porosome, which has been implicated in the rapid closing and opening of the complex^80^. ©Bhanu Jena.

**Figure 6:**
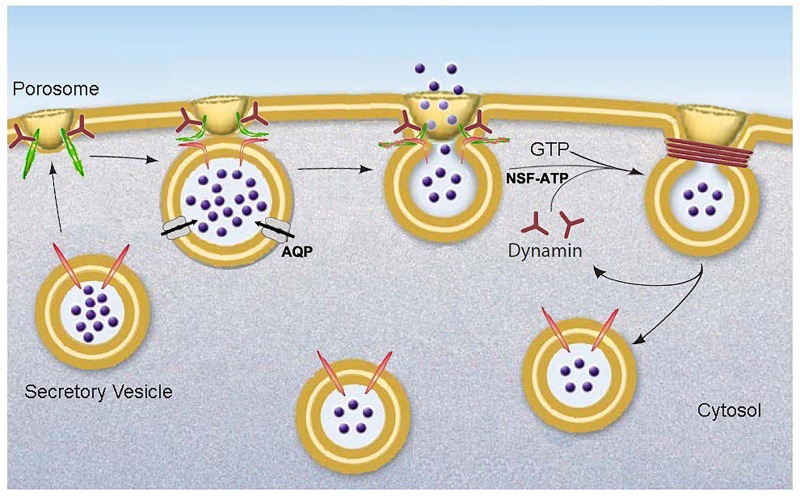
Schematic drawing depicting the presence and increased association of dynamin with the porosome complex following stimulation of neurotransmitter release12, which may be similar in the mucin-secreting Calu-3 cell Following stimulation of secretion, synaptic vesicles would dock at the porosome base, develop intravesicular pressure via active transport of water through water channels or aquaporins (AQP) at the vesicle membrane, transiently fuse at the porosome base via SNAREs and calcium, and expel neurotransmitters. After secretion, NSF an ATPase, and dynamin a GTPase, would work synchronously to disassembly t-/v-SNARE complexes and fission the neck of fused vesicles at the porosome base respectively. By this mechanism, partially empty vesicles could go through multiple rounds of docking-fusion-expulsion-dissociation. Unlike protein and peptide containing vesicles, synaptic vesicles have neurotransmitter transporters at the vesicle membrane to rapidly refill vesicles^12^. In case of the Calu-3 cell, once mucin containing vesicles empty, they may recycle via the endosome or lysosomal pathway. ©Bhanu Jena.

It is well established that cup-shaped macro-molecular lipoprotein structures called *porosomes* are secretory portals embedded in the cell plasma membrane in cells, where membrane-bound secretory vesicles transiently dock and fuse to expel intravesicular contents during secretion^1-10^. Porosomes have been isolated from a number of cells, including the exocrine pancreas^5,6^ (**Figure 1**), neurons^3^ (**Figure 2**), and in the mucin-secreting human airway epithelial cell line Calu-3 (**Figure 3, Figure 4**)^11^. The morphology, composition, and reconstitution of porosomes in the exocrine pancreas (**Figure 5, Figure 6**) and in neurons are well documented^2-6,8-12^, and the 3D contour map of the assembly of proteins within the structure has also been determined in great detail^10^. This new understanding of the secretory machinery in cells now provides a platform to address diseases resulting from secretory defects. The structure, and composition of the porosome complex in Calu-3 cells expressing cystic fibrosis (CF) transmembrane conductance regulator (CFTR) has been determined for the first time^11^, with promise to help better understand cystic fibrosis. CFTR is a plasma membrane chloride selective cyclic AMP-activated ion channel, localized at the apical membrane of secretory epithelial cells, including the conducting airways^13^. Besides mediating the secretion of Cl^-^, CFTR also regulates several other transport proteins, including K^+^ channels, aquaporin water channels, anion exchangers, the membrane fusion protein syntaxin-1A, and sodium bicarbonate transporters^14-24^. Accordingly, studies show that CFTR and its associated proteins are present in large macromolecular signaling complexes via scaffolding proteins containing PDZ domains^13,25-27^. The C-terminus of CFTR in humans contains the sequence Asp-Thr-Arg-Leu, that mediate binding to several PDZ domain proteins^13^. For example, ezrin and moesin present in the Calu-3 porosome complex^11^are also known CFTR-PDZ binding protein^13^. In addition, CFTR has several other regions that mediate protein-protein interactions, such as a domain at its N-terminus that binds to syntaxin-1A and SNAP-23^15,23^. CFTR also contains a protein phosphatase-2A (PP2A)-binding, and an AMP kinase (AMPK)-binding domain^12^. Similarly, CFTR has a regulatory domain that is a substrate to both protein kinase A (PKA) and C (PKC)^28^. These interactions facilitate CFTR to form large CFTR-associated macromolecular signaling complexes at the plasma membrane. CF as a disease was first identified as cysts observed in the pancreas and the highly viscous mucus found in the lung of patients. However, since discovery that these observed defects are a result of a dysfunction of the CFTR chloride channel^29,30^, there has been little progress in our understanding of the link between CFTR dysfunction and the secretion of such highly viscous mucin in the lung of CF patients^31^. The surface of the airways is coated with a thin film of mucous composed of essentially mucin, salt, proteases, antioxidants, and antibodies^31,32^. Mucin lubricates, trap foreign particles and pathogens, and assists in the clearance of foreign particles from the airways via ciliary transport^31,32^. A key property of mucus is its appropriate viscosity that enables its movement by the underlying cilia. Secretion of more viscous mucus disallows its proper transport, resulting in chronic and fatal airways disease such as CF^32^. Similar to other secretory cells that undergo secretory vesicle volume increase during secretion^33-45^, goblet cells of the airways epithelia that store mucin in a dehydrated state within membrane-bound secretory granules are no exception. Since vesicle swelling is a requirement for cell secretion^41^, and both ion channels and water channels or aquaporins regulate this process^42,44^, altered chloride transport would impair secretory vesicle hydration and optimal release. Furthermore, recent studies in mice lacking functional CFTR^31^ showed that these animals secrete highly viscous mucous that adhered to the epithelium. Since CFTR is known to interact with syntaxin-1A, chloride channel CLC-3, and aquaporins^14-26^, which are components of the porosome complex^1,2,5-7,46^, the possible interactions between CFTR and the porosome in goblet cells was hypothesized and tested in a recent study^11^. Results from the study demonstrate the presence of approximately 100 nm in size porosomes and microvilli at the surface of the plasma membrane in Calu-3 cells (**Figure 1, Figure 2**)^11^.

The t-SNARE SNAP-25 specific antibody conjugated to protein A-sepharose® has been utilized to isolate the porosome complex from Calu-3 cells^11^. For each immunoisolation, 1 mg of Triton-Lubrol-solubilized Calu-3 cells was used. The Triton/Lubrol solubilization buffer contained 0.5% Lubrol, 0.5% Triton X-100, 1 mM benzamidine, 5 mM Mg-ATP, and 5 mM EDTA in PBS at pH 7.5, supplemented with protease inhibitor mix (Sigma, St. Louis, MO). Ten micrograms of SNAP-25 antibody conjugated to the protein A-sepharose® were incubated with the 1 mg of the solubilized cells for 1 h at room temperature followed by three washes of 10 volumes of wash buffer (500 mM NaCl, 10 mM Tris, 2 mM EDTA, pH 7.5). The immunoprecipitated porosome attached to the immunosepharose beads was eluted using low pH buffer (pH 3.5) to dissociate the complex from the antibody bound to the bead, and the eluted sample immediately returned to neutral pH and stored at -80 degrees^11^. A combination of proteomics, Western blot analysis, and immunocytochemistry, were all used to determine the composition and distribution of the porosome complex in Calu-3 cells^11^. Proteomic analysis of isolated Calu-3 porosomes using mass spectrometry demonstrate the presence of CFTR as well as several proteins found in the neuronal porosome complex, including Syntaxin-1A, actin, rabs, heterotrimeric G-protein, and the GTPase activating protein GAP (Table I)^11^. Immunoblot analysis (**Figure 3**) of the isolated Calu-3 porosome complex, and immunocytochemistry (**Figure 4**) further confirms CFTR association with the porosome complex, reflecting important implication of CFTR in both normal mucus secretion in the airway epithelium in health, and in the impaired state in CF disease. In the past two decades, employing a combination of approaches including AFM, biochemistry, molecular biology, electrophysiology, EM, mass spectrometry, SAXS analysis, and database searches such as STRING 9.1 of known functional and predicted protein-protein interactions, further structural details of the porosome complex have been determined^1-12^. Although great progress have been made in our understanding of the porosome, of Ca^+2^ and SNARE-mediated membrane fusion^47-76^, and on secretory vesicle volume regulation required for regulated fractional release of intravesicular contents^35-45^ during cell secretion, a molecular level understanding of porosome-mediated secretion in mucin-secreting cells remains to be determined. Therefore, a clear understanding of the porosome in mucin secreting in Calu-3 cells, and the role of CFTR in porosome-mediated mucin secretion is critical in revealing how mucin secretion is precisely regulated.

## 2. Porosome in Calu-3 cell

High resolution imaging using atomic force microscopy (AFM) (**Figure 1**) reveal in great detail the surface topology of Calu-3 cells, demonstrate the presence of approximately 102 nm in diameter porosome openings (mean ± SEM; 102.4 nm ± 3.0 nm), and 96 nm thick (mean ± SEM; 96 nm ± 3.3 nm) microvilli, at the cell plasma membrane. Nearly the entire cell surface is covered with the microvilli, with interspersed porosome openings. In certain areas of the cell surface devoid of microvilli or porosome openings, cytoskeletal structures underlying the cell plasma membrane are observed (**Figure 1** [B])^11^. Transmission electron microscopy (TEM) performed on Calu-3 cells confirms the AFM results (**Figure 2**), demonstrating the presence of dense microvilli (**Figure 2** [A]), and cup-shaped porosomes (**Figure 2** [B-D]) at the cell plasma membrane. Immunoisolated Calu-3 porosome complexes demonstrate a particle size of approximately 300 nm (**Figure 3** [A]) using photon correlation spectroscopy (PCS), possibly a result of trimerization in their isolated state in suspension^11^. Immunoblot analysis of isolated Calu-3 porosome complex (**Figure 3** [B]), demonstrate the co-association of CFTR with the porosome complex that contains among other proteins Gai3, actin, and vimentin (**Figure 3** [C]). Similarly, when CFTR is immunoisolated from solubilized Calu-3 cells, the porosome complex is co-immunoisolated with CFTR, as demonstrated by the presence of porosome-associated proteins Syntaxin-1A, SNAP-25, SNAP-23, actin, and vimentin (**Figure 3** [D]). Mass spectrometry on the immunoisolated porosome complex from Calu-3 cells, demonstrates the presence of various porosome-associated proteins (found in the neuronal and pancreatic porosome complexes) as well as CFTR^11^. Furthermore, mass spectrometry results confirm the interaction between CFTR and the porosome complex in Calu-3 cells, as determined using imunoprecipitation and immunoblot analysis. Using the STRING 9.1 database search^77^ similar to the neuronal porosome complex^12^, two clusters of protein-protein interactions within the mucin-secreting Calu-3 porosome have been identified (**Figure 4**). The cluster to the top-right in **Figure 4**, represent primarily cytoskeletal and signaling proteins, whereas the bottom-left cluster represents proteins that are primarily involved in membrane fusion, including SNAREs, ion channels and CFTR. Therefore the bottom cluster is located at the porosome base facing the cytosol where mucin-containing vesicles dock and fuse, and the top cluster at the porosome opening to the outside. Therefore in these studies, the porosome proteome in human airways epithelia has been determined. The interaction between CFTR and the porosome complex in the human airways epithelia is further demonstrated. The possible regulation by CFTR on the quality of mucus secretion via the porosome complex at the cell plasma membrane is hypothesized. These new findings will facilitate understanding of CFTR-porosome interactions influencing mucus secretion, and provide critical insights into the etiology of CF disease.

In view of this, an integrated approach is being used to characterize the molecular architecture of the mucin-secreting porosome complex of the human airways epithelia cell line Calu-3; determine the distribution of CFTR and its interaction with proteins and lipids within the mucin-secreting porosome complex; characterize the molecular architecture of the mucin-secreting porosome; and build and test a functional architectural model to determine how SNAREs, lipids, and calcium, establish continuity between the secretory vesicle membrane and the porosome. These studies will allow a molecular understanding of the porosome function in mucin secretion, and the role of CFTR in the process.

## 3. Ongoing studies

Among the 34 core porosome proteins in mucin-secreting Calu-3 cells, are included CFTR, actin, vimentin, annexin, filamin, Gai3, tubulin, syntaxin-1A, profiling, ezrin, spectrin, chloride channels CLC-1 and CLC-3, rab1A and rab3A, myosin, SNAP-25, and the ADP-ribosylation factor ARF3^11^. It is anticipated that due to the nature of proteome studies and the dynamics of porosomes^12^that this initial analysis includes most of the core porosome proteins, but not all the peripheral proteins associated with the complex. The STRING 9.1^77^ database search utilizing known physical and functional associations between proteins suggests additional candidates likely involved in protein-protein interactions within the Calu-3 porosome complex (**Figure 4**). Similarly, preliminary lipidomic studies using lipid overlay assays on isolated Calu-3 porosome complex, demonstrate the enriched presence of PA and PIP2. Furthermore, interactions of PA and other polyphosphoinositides with syntaxin-1A, and their involvement in cell secretion have previously been reported^78^. These observations indicate the importance of lipid interactions in the structure and function of the porosome. Based on this information, and since mucin containing vesicles dock at the porosome base, and the t-SNARE syntaxin-1A and SNAP-25 associated with CFTR, rab3C, CLC3, and SNAP91 are among other proteins present at the porosome base (**Figure 4**), it could be surmised that PA and PIP2 together with these proteins are present at the porosome base. The protein cluster composed of cytoskeletal and signaling proteins on the other hand, is likely associated with the porosome opening to the outside of the cell, regulating dilation of the porosome opening during mucin secretion. Since in the presence of the actin depolymerizing agent cytocholasine, there is loss of mucin secretion (unpublished observation) as in the case of the exocrine pancreas^7^ or growth hormone secreting cells^1^, further supports the presence of signaling and motor proteins at the porosome opening, that regulate dilation of the porosome opening and mucin secretion.

Unlike individual proteins or lipids, determination of the atomic structure of such dynamic macromolecular lipoprotein complexes such as the porosome, poses a difficult challenge, requiring the use of severalexperimental and computational approaches to maximize resolution and accuracy. Although recently the isolated Calu-3 porosome has been functionally reconstituted as in case of the exocrine pancreas or neurons, further functional reconstitution studies are under way using the established lipid bilayer EPC9 system^6^. These experiments further determine if the entire porosome complex has been isolated prior to determination of its composition and molecular structure-function. Electron microscopy (EM) especially single particle cryo electron tomography, small angle X-ray solution scattering (SAXS), supra resolution microscopy (SRM), and AFM analyses are being used and complemented by techniques from structural mass spectrometry and proteomics to obtain molecular details of the mucin-secreting porosome structure. The mass spectrometry studies include subunit stoichiometry, interacting subunits, and site of contact between subunits. Changes to porosome subunit composition and subunit interactions during the secretory process are being studies using these approaches. CXL-MS and multiple quantitative mass spectrometry techniques are being utilized to determination details of the protein-protein interactions within the native mucin-secreting porosome complex, which is central to building a structural model of the complex for a molecular understanding of its structure-function.

New and recently developed crosslinkers^79^ combined with tandem mass spectrometry are being carried out, which will provide identities of interacting subunits and provide the identities of specific residues crosslinked both between and within subunits in the porosome complex. Results from these studies will provide information on interaction domains and distance constraints on protein structures. Quantitative mass spectrometry using iTRAQ are also being carried out, which will provide additional information on changes in porosome subunits composition and dynamics, as a function of the secretion status of the organelle. Immuno-AFM^5^, immuno–EM, and SAXS^80^ (**Figure 5**) studies on isolated Calu-3 porosomes as in porosomes of the exocrine pancreas and neurons, are being performed to determine the distribution of some of the major proteins within the complex. Similar to studies using SRM on the nuclear pore complex^81^, SRM is being employed to obtain additional information on the structure of the mucin-secreting porosome complex. Finally, computational approaches are being employed, such as coarse-grain molecular docking studies^82-97^, homology modeled interactions^98-100^, and fitting of known atomic structures of protein-protein interactions and complexes^101-108^. It is becoming increasingly clear that the ultrastructural and mass spectrometry methods show promise in providing complementary information and the high degree of cross-validation required to build an accurate structural model of the mucin-secreting porosome complex. Collectively, the outlined studies briefly discussed here will enable an understanding at the molecular level, the elegant mechanism of porosome-mediated secretion (**Figure 6**) in Calu-3 and other cells.

## KEY POINTS

- **Mucin-secreting porosomes are 100 nm cup-shaped secretory portal at the plasma membrane; the porosome has been isolated from Calu-3 cells and its proteome determined**.
- **Porosome structure has been determined using EM and AFM**.
- **Distribution of lipids and various proteins especially CFTR within the porosome complex is under way**.

## References

[1] Cho Sang-Joon, Jeftinija Ksenija, Glavaski Aleksandra, Jeftinija Srdija, Jena Bhanu P, Anderson Lloyd L (2002). Structure and dynamics of the fusion pores in live GH-secreting cells revealed using atomic force microscopy.. Endocrinology.

[2] Cho Sang-Joon, Quinn Anthony S, Stromer Marvin H, Dash Sudhansu, Cho Jinah, Taatjes Douglas J, Jena Bhanu P (2002). Structure and dynamics of the fusion pore in live cells.. Cell biology international.

[3] Cho Won Jin, Jeremic Aleksandar, Rognlien Kathy T, Zhvania Mzia G, Lazrishvili Ilia, Tamar Bikashvili, Jena Bhanu P (2004). Structure, isolation, composition and reconstitution of the neuronal fusion pore.. Cell biology international.

[4] Cho Won Jin, Jeremic Aleksandar, Jin Huan, Ren Gang, Jena Bhanu P (2007). Neuronal fusion pore assembly requires membrane cholesterol.. Cell biology international.

[5] Jena Bhanu P, Cho Sang-Joon, Jeremic Aleksandar, Stromer Marvin H, Abu-Hamdah Rania (2003). Structure and composition of the fusion pore.. Biophysical journal.

[6] Jeremic Aleksandar, Kelly Marie, Cho Sang-Joon, Stromer Marvin H, Jena Bhanu P (2003). Reconstituted fusion pore.. Biophysical journal.

[7] Schneider S W, Sritharan K C, Geibel J P, Oberleithner H, Jena B P (1997). Surface dynamics in living acinar cells imaged by atomic force microscopy: identification of plasma membrane structures involved in exocytosis.. Proceedings of the National Academy of Sciences of the United States of America.

[8] Jena Bhanu P (2005). Molecular machinery and mechanism of cell secretion.. Experimental biology and medicine (Maywood, N.J.).

[9] Jena Bhanu P (2007). Secretion machinery at the cell plasma membrane.. Current opinion in structural biology.

[10] Cho W J, Ren G, Jena B P (2008). EM 3D contour maps provide protein assembly at the nanoscale within the neuronal porosome complex.. Journal of microscopy.

[11] Hou Xia, Lewis Kenneth T, Wu Qingtian, Wang Sunxi, Chen Xuequn, Flack Amanda, Mao Guangzhao, Taatjes Douglas J, Sun Fei, Jena Bhanu P (2014). Proteome of the porosome complex in human airway epithelia: interaction with the cystic fibrosis transmembrane conductance regulator (CFTR).. Journal of proteomics.

[12] Lee Jin-Sook, Jeremic Aleksandar, Shin Leah, Cho Won Jin, Chen Xuequn, Jena Bhanu P (2012). Neuronal porosome proteome: Molecular dynamics and architecture.. Journal of proteomics.

[13] Guggino William B, Stanton Bruce A (2006). New insights into cystic fibrosis: molecular switches that regulate CFTR.. Nature reviews. Molecular cell biology.

[14] Schwiebert E M, Egan M E, Hwang T H, Fulmer S B, Allen S S, Cutting G R, Guggino W B (1995). CFTR regulates outwardly rectifying chloride channels through an autocrine mechanism involving ATP.. Cell.

[15] Naren A P, Nelson D J, Xie W, Jovov B, Pevsner J, Bennett M K, Benos D J, Quick M W, Kirk K L (1997). Regulation of CFTR chloride channels by syntaxin and Munc18 isoforms.. Nature.

[16] Naren Anjaparavanda P, Cobb Bryan, Li Chunying, Roy Koushik, Nelson David, Heda Ghanshyam D, Liao Jie, Kirk Kevin L, Sorscher Eric J, Hanrahan John, Clancy John P (2003). A macromolecular complex of beta 2 adrenergic receptor, CFTR, and ezrin/radixin/moesin-binding phosphoprotein 50 is regulated by PKA.. Proceedings of the National Academy of Sciences of the United States of America.

[17] Stutts M J, Rossier B C, Boucher R C (1997). Cystic fibrosis transmembrane conductance regulator inverts protein kinase A-mediated regulation of epithelial sodium channel single channel kinetics.. The Journal of biological chemistry.

[18] Shumaker Holli, Amlal Hassane, Frizzell Raymond, Ulrich Charles D., Soleimani Manoocher (1999). CFTR drives Na+- n HCO 3 − cotransport in pancreatic duct cells: a basis for defective HCO 3 − secretion in CF. American Journal of Physiology-Cell Physiology.

[19] Ji H L, Chalfant M L, Jovov B, Lockhart J P, Parker S B, Fuller C M, Stanton B A, Benos D J (2000). The cytosolic termini of the beta- and gamma-ENaC subunits are involved in the functional interactions between cystic fibrosis transmembrane conductance regulator and epithelial sodium channel.. The Journal of biological chemistry.

[20] Jiang Q, Li J, Dubroff R, Ahn Y J, Foskett J K, Engelhardt J, Kleyman T R (2000). Epithelial sodium channels regulate cystic fibrosis transmembrane conductance regulator chloride channels in Xenopus oocytes.. The Journal of biological chemistry.

[21] Sun F, Hug M J, Bradbury N A, Frizzell R A (2000). Protein kinase A associates with cystic fibrosis transmembrane conductance regulator via an interaction with ezrin.. The Journal of biological chemistry.

[22] Cheung K H, Leung C T, Leung G P H, Wong P Y D (2003). Synergistic effects of cystic fibrosis transmembrane conductance regulator and aquaporin-9 in the rat epididymis.. Biology of reproduction.

[23] Ganeshan Radhika, Di Anke, Nelson Deborah J, Quick Michael W, Kirk Kevin L (2003). The interaction between syntaxin 1A and cystic fibrosis transmembrane conductance regulator Cl- channels is mechanistically distinct from syntaxin 1A-SNARE interactions.. The Journal of biological chemistry.

[24] Ko Shigeru B H, Zeng Weizhong, Dorwart Michael R, Luo Xiang, Kim Kil Hwan, Millen Linda, Goto Hidemi, Naruse Satoru, Soyombo Abigail, Thomas Philip J, Muallem Shmuel (2004). Gating of CFTR by the STAS domain of SLC26 transporters.. Nature cell biology.

[25] Li Chunying, Roy Koushik, Dandridge Keanna, Naren Anjaparavanda P (2004). Molecular assembly of cystic fibrosis transmembrane conductance regulator in plasma membrane.. The Journal of biological chemistry.

[26] Yoo Dana, Flagg Thomas P, Olsen Olav, Raghuram Viswanathan, Foskett J Kevin, Welling Paul A (2004). Assembly and trafficking of a multiprotein ROMK (Kir 1.1) channel complex by PDZ interactions.. The Journal of biological chemistry.

[27] Guggino William B (2004). The cystic fibrosis transmembrane regulator forms macromolecular complexes with PDZ domain scaffold proteins.. Proceedings of the American Thoracic Society.

[28] Riordan John R (2005). Assembly of functional CFTR chloride channels.. Annual review of physiology.

[29] Quinton P M (1983). Chloride impermeability in cystic fibrosis.. Nature.

[30] Riordan J R, Rommens J M, Kerem B, Alon N, Rozmahel R, Grzelczak Z, Zielenski J, Lok S, Plavsic N, Chou J L (1989). Identification of the cystic fibrosis gene: cloning and characterization of complementary DNA.. Science (New York, N.Y.).

[31] Gustafsson Jenny K, Ermund Anna, Ambort Daniel, Johansson Malin E V, Nilsson Harriet E, Thorell Kaisa, Hebert Hans, Sjövall Henrik, Hansson Gunnar C (2012). Bicarbonate and functional CFTR channel are required for proper mucin secretion and link cystic fibrosis with its mucus phenotype.. The Journal of experimental medicine.

[32] Knowles Michael R, Boucher Richard C (2002). Mucus clearance as a primary innate defense mechanism for mammalian airways.. The Journal of clinical investigation.

[33] Hovenberg H W, Davies J R, Carlstedt I (1996). Different mucins are produced by the surface epithelium and the submucosa in human trachea: identification of MUC5AC as a major mucin from the goblet cells.. The Biochemical journal.

[34] Wickström C, Davies J R, Eriksen G V, Veerman E C, Carlstedt I (1998). MUC5B is a major gel-forming, oligomeric mucin from human salivary gland, respiratory tract and endocervix: identification of glycoforms and C-terminal cleavage.. The Biochemical journal.

[35] Finkelstein A, Zimmerberg J, Cohen F S (1986). Osmotic swelling of vesicles: its role in the fusion of vesicles with planar phospholipid bilayer membranes and its possible role in exocytosis.. Annual review of physiology.

[36] Holz R W (1986). The role of osmotic forces in exocytosis from adrenal chromaffin cells.. Annual review of physiology.

[37] Fernandez J M, Villalón M, Verdugo P (1991). Reversible condensation of mast cell secretory products in vitro.. Biophysical journal.

[38] Monck J R, Oberhauser A F, Alvarez de Toledo G, Fernandez J M (1991). Is swelling of the secretory granule matrix the force that dilates the exocytotic fusion pore?. Biophysical journal.

[39] Jena B P, Schneider S W, Geibel J P, Webster P, Oberleithner H, Sritharan K C (1997). Gi regulation of secretory vesicle swelling examined by atomic force microscopy.. Proceedings of the National Academy of Sciences of the United States of America.

[40] Cho Sang-Joon, Sattar A K M Abdus, Jeong Eun-Hwan, Satchi Mylan, Cho Jin Ah, Dash Sudhansu, Mayes Mary Sue, Stromer Marvin H, Jena Bhanu P (2002). Aquaporin 1 regulates GTP-induced rapid gating of water in secretory vesicles.. Proceedings of the National Academy of Sciences of the United States of America.

[41] Kelly Marie L, Cho Won Jin, Jeremic Aleksandar, Abu-Hamdah Rania, Jena Bhanu P (2004). Vesicle swelling regulates content expulsion during secretion.. Cell biology international.

[42] Kelly Marie L, Abu-Hamdah Rania, Jeremic Aleksandar, Cho Sang-Joon, Ilie Alina-Elena, Jena Bhanu P (2005). Patch clamped single pancreatic zymogen granules: direct measurements of ion channel activities at the granule membrane.. Pancreatology : official journal of the International Association of Pancreatology (IAP) ... [et al.].

[43] Lee Jin-Sook, Cho Won Jin, Shin Leah, Jena Bhanu P (2010). Involvement of cholesterol in synaptic vesicle swelling.. Experimental biology and medicine (Maywood, N.J.).

[44] Shin Leah, Basi Nirukti, Jeremic Aleksandar, Lee Jin-Sook, Cho Won Jin, Chen Zhihui, Abu-Hamdah Rania, Oupicky David, Jena Bhanu P (2010). Involvement of vH(+)-ATPase in synaptic vesicle swelling.. Journal of neuroscience research.

[45] Chen Zhi Hui, Lee Jin-Sook, Shin Leah, Cho Won Jin, Jena Bhanu P (2011). Involvement of β-adrenergic receptor in synaptic vesicle swelling and implication in neurotransmitter release.. Journal of cellular and molecular medicine.

[46] Cho S-J, Wakade A, Pappas G D, Jena B P (2002). New structure involved in transient membrane fusion and exocytosis.. Annals of the New York Academy of Sciences.

[47] Trimble W S, Cowan D M, Scheller R H (1988). VAMP-1: a synaptic vesicle-associated integral membrane protein.. Proceedings of the National Academy of Sciences of the United States of America.

[48] Bennett M K, Calakos N, Scheller R H (1992). Syntaxin: a synaptic protein implicated in docking of synaptic vesicles at presynaptic active zones.. Science (New York, N.Y.).

[49] Oyler G A, Higgins G A, Hart R A, Battenberg E, Billingsley M, Bloom F E, Wilson M C (1989). The identification of a novel synaptosomal-associated protein, SNAP-25, differentially expressed by neuronal subpopulations.. The Journal of cell biology.

[50] Weber T, Zemelman B V, McNew J A, Westermann B, Gmachl M, Parlati F, Söllner T H, Rothman J E (1998). SNAREpins: minimal machinery for membrane fusion.. Cell.

[51] Sutton R B, Fasshauer D, Jahn R, Brunger A T (1998). Crystal structure of a SNARE complex involved in synaptic exocytosis at 2.4 A resolution.. Nature.

[52] Cho Sang-Joon, Kelly Marie, Rognlien Katherine T, Cho Jin Ah, Hörber J K Heinrich, Jena Bhanu P (2002). SNAREs in opposing bilayers interact in a circular array to form conducting pores.. Biophysical journal.

[53] Jeremic Aleksandar, Kelly Marie, Cho Jin Ah, Cho Sang-Joon, Horber J K Heinrich, Jena Bhanu P (2004). Calcium drives fusion of SNARE-apposed bilayers.. Cell biology international.

[54] Jeremic A (2002). Membrane fusion: what may transpire at the atomic level. Journal of Biological Physics and Chemistry.

[55] Cho Won Jin, Jeremic Aleksandar, Jena Bhanu P. (2005). Size of Supramolecular SNARE Complex:  Membrane-Directed Self-Assembly. Journal of the American Chemical Society.

[56] Jeremic Aleksandar, Quinn Anthony S, Cho Won Jin, Taatjes Douglas J, Jena Bhanu P (2006). Energy-dependent disassembly of self-assembled SNARE complex: observation at nanometer resolution using atomic force microscopy.. Journal of the American Chemical Society.

[57] Cook Jeremy D, Cho Won Jin, Stemmler Timothy L, Jena Bhanu P (2008). Circular dichroism (CD) spectroscopy of the assembly and disassembly of SNAREs: The proteins involved in membrane fusion in cells.. Chemical physics letters.

[58] Shin Leah, Cho Won Jin, Cook Jeremy D, Stemmler Timothy L, Jena Bhanu P (2010). Membrane lipids influence protein complex assembly-disassembly.. Journal of the American Chemical Society.

[59] Potoff Jeffrey J, Issa Zeena, Manke Charles W, Jena Bhanu P (2008). Ca2+-dimethylphosphate complex formation: providing insight into Ca2+-mediated local dehydration and membrane fusion in cells.. Cell biology international.

[60] Cho Won Jin, Lee Jin-Sook, Zhang Lei, Ren Gang, Shin Leah, Manke Charles W, Potoff Jeffrey, Kotaria Nato, Zhvania Mzia G, Jena Bhanu P (2011). Membrane-directed molecular assembly of the neuronal SNARE complex.. Journal of cellular and molecular medicine.

[61] Misura K M, Scheller R H, Weis W I (2000). Three-dimensional structure of the neuronal-Sec1-syntaxin 1a complex.. Nature.

[62] Sudhof Thomas C (2004). The synaptic vesicle cycle.. Annual review of neuroscience.

[63] Portis A, Newton C, Pangborn W, Papahadjopoulos D (1979). Studies on the mechanism of membrane fusion: evidence for an intermembrane Ca2+-phospholipid complex, synergism with Mg2+, and inhibition by spectrin.. Biochemistry.

[64] Pobbati Ajaybabu V, Stein Alexander, Fasshauer Dirk (2006). N- to C-terminal SNARE complex assembly promotes rapid membrane fusion.. Science (New York, N.Y.).

[65] Jahn Reinhard, Scheller Richard H (2006). SNAREs--engines for membrane fusion.. Nature reviews. Molecular cell biology.

[66] Shen Jingshi, Tareste David C, Paumet Fabienne, Rothman James E, Melia Thomas J (2007). Selective activation of cognate SNAREpins by Sec1/Munc18 proteins.. Cell.

[67] Martens Sascha, Kozlov Michael M, McMahon Harvey T (2007). How synaptotagmin promotes membrane fusion.. Science (New York, N.Y.).

[68] Wickner William, Schekman Randy (2008). Membrane fusion.. Nature structural & molecular biology.

[69] Chapman Edwin R (2008). How does synaptotagmin trigger neurotransmitter release?. Annual review of biochemistry.

[70] Hui Enfu, Johnson Colin P, Yao Jun, Dunning F Mark, Chapman Edwin R (2009). Synaptotagmin-mediated bending of the target membrane is a critical step in Ca(2+)-regulated fusion.. Cell.

[71] Südhof Thomas C, Rothman James E (2009). Membrane fusion: grappling with SNARE and SM proteins.. Science (New York, N.Y.).

[72] Stein Alexander, Weber Gert, Wahl Markus C, Jahn Reinhard (2009). Helical extension of the neuronal SNARE complex into the membrane.. Nature.

[73] Brunger Axel T, Weninger Keith, Bowen Mark, Chu Steven (2009). Single-molecule studies of the neuronal SNARE fusion machinery.. Annual review of biochemistry.

[74] Südhof Thomas C, Rizo Josep (2011). Synaptic vesicle exocytosis.. Cold Spring Harbor perspectives in biology.

[75] Yao Jun, Gaffaney Jon D, Kwon Sung E, Chapman Edwin R (2011). Doc2 is a Ca2+ sensor required for asynchronous neurotransmitter release.. Cell.

[76] Diao Jiajie, Ishitsuka Yuji, Bae Woo-Ri (2011). Single-molecule FRET study of SNARE-mediated membrane fusion.. Bioscience reports.

[77] Szklarczyk Damian, Franceschini Andrea, Kuhn Michael, Simonovic Milan, Roth Alexander, Minguez Pablo, Doerks Tobias, Stark Manuel, Muller Jean, Bork Peer, Jensen Lars J, von Mering Christian (2011). The STRING database in 2011: functional interaction networks of proteins, globally integrated and scored.. Nucleic acids research.

[78] Lam Alice D, Tryoen-Toth Petra, Tsai Bill, Vitale Nicolas, Stuenkel Edward L (2008). SNARE-catalyzed fusion events are regulated by Syntaxin1A-lipid interactions.. Molecular biology of the cell.

[79] Clifford-Nunn Billy, Showalter H D Hollis, Andrews Philip C (2012). Quaternary diamines as mass spectrometry cleavable crosslinkers for protein interactions.. Journal of the American Society for Mass Spectrometry.

[80] Kovari Ladislau C, Brunzelle Joseph S, Lewis Kenneth T, Cho Won Jin, Lee Jin-Sook, Taatjes Douglas J, Jena Bhanu P (2014). X-ray solution structure of the native neuronal porosome-synaptic vesicle complex: Implication in neurotransmitter release.. Micron (Oxford, England : 1993).

[81] Szymborska Anna, de Marco Alex, Daigle Nathalie, Cordes Volker C, Briggs John A G, Ellenberg Jan (2013). Nuclear pore scaffold structure analyzed by super-resolution microscopy and particle averaging.. Science (New York, N.Y.).

[82] Gray Jeffrey J, Moughon Stewart E, Kortemme Tanja, Schueler-Furman Ora, Misura Kira M S, Morozov Alexandre V, Baker David (2003). Protein-protein docking predictions for the CAPRI experiment.. Proteins.

[83] Smith Graham R, Sternberg Michael J E (2002). Prediction of protein-protein interactions by docking methods.. Current opinion in structural biology.

[84] Janin Joël, Henrick Kim, Moult John, Eyck Lynn Ten, Sternberg Michael J E, Vajda Sandor, Vakser Ilya, Wodak Shoshana J (2003). CAPRI: a Critical Assessment of PRedicted Interactions.. Proteins.

[85] Schneidman-Duhovny Dina, Inbar Yuval, Polak Vladimir, Shatsky Maxim, Halperin Inbal, Benyamini Hadar, Barzilai Adi, Dror Oranit, Haspel Nurit, Nussinov Ruth, Wolfson Haim J. (2003). Taking geometry to its edge: Fast unbound rigid (and hinge-bent) docking. Proteins: Structure, Function, and Genetics.

[86] Katchalski-Katzir E, Shariv I, Eisenstein M, Friesem A A, Aflalo C, Vakser I A (1992). Molecular surface recognition: determination of geometric fit between proteins and their ligands by correlation techniques.. Proceedings of the National Academy of Sciences of the United States of America.

[87] Gabb H A, Jackson R M, Sternberg M J (1997). Modelling protein docking using shape complementarity, electrostatics and biochemical information.. Journal of molecular biology.

[88] Moont G, Sternberg MJ (2001). Modeling protein-protein and protein-DNA docking.

[89] Jackson R M, Gabb H A, Sternberg M J (1998). Rapid refinement of protein interfaces incorporating solvation: application to the docking problem.. Journal of molecular biology.

[90] Vakser I A (1995). Protein docking for low-resolution structures.. Protein engineering.

[91] Mandell J G, Roberts V A, Pique M E, Kotlovyi V, Mitchell J C, Nelson E, Tsigelny I, Ten Eyck L F (2001). Protein docking using continuum electrostatics and geometric fit.. Protein engineering.

[92] Chen Rong, Li Li, Weng Zhiping (2003). ZDOCK: an initial-stage protein-docking algorithm.. Proteins.

[93] Ritchie D W, Kemp G J (2000). Protein docking using spherical polar Fourier correlations.. Proteins.

[94] Fernández-Recio Juan, Totrov Maxim, Abagyan Ruben (2002). Soft protein-protein docking in internal coordinates.. Protein science : a publication of the Protein Society.

[95] Gray Jeffrey J, Moughon Stewart, Wang Chu, Schueler-Furman Ora, Kuhlman Brian, Rohl Carol A, Baker David (2003). Protein-protein docking with simultaneous optimization of rigid-body displacement and side-chain conformations.. Journal of molecular biology.

[96] Gabdoulline R R, Wade R C (2001). Protein-protein association: investigation of factors influencing association rates by brownian dynamics simulations.. Journal of molecular biology.

[97] Fitzjohn Paul W, Bates Paul A (2003). Guided docking: first step to locate potential binding sites.. Proteins.

[98] Aloy Patrick, Russell Robert B (2002). Interrogating protein interaction networks through structural biology.. Proceedings of the National Academy of Sciences of the United States of America.

[99] Aloy Patrick, Böttcher Bettina, Ceulemans Hugo, Leutwein Christina, Mellwig Christian, Fischer Susanne, Gavin Anne-Claude, Bork Peer, Superti-Furga Giulio, Serrano Luis, Russell Robert B (2004). Structure-based assembly of protein complexes in yeast.. Science (New York, N.Y.).

[100] Pieper Ursula, Webb Benjamin M, Dong Guang Qiang, Schneidman-Duhovny Dina, Fan Hao, Kim Seung Joong, Khuri Natalia, Spill Yannick G, Weinkam Patrick, Hammel Michal, Tainer John A, Nilges Michael, Sali Andrej (2014). ModBase, a database of annotated comparative protein structure models and associated resources.. Nucleic acids research.

[101] Martí-Renom M A, Stuart A C, Fiser A, Sánchez R, Melo F, Sali A (2000). Comparative protein structure modeling of genes and genomes.. Annual review of biophysics and biomolecular structure.

[102] Volkmann N, Hanein D, Ouyang G, Trybus K M, DeRosier D J, Lowey S (2000). Evidence for cleft closure in actomyosin upon ADP release.. Nature structural biology.

[103] Roseman A M (2000). Docking structures of domains into maps from cryo-electron microscopy using local correlation.. Acta crystallographica. Section D, Biological crystallography.

[104] Wriggers W, Birmanns S (2001). Using situs for flexible and rigid-body fitting of multiresolution single-molecule data.. Journal of structural biology.

[105] Ceulemans Hugo, Russell Robert B (2004). Fast Fitting of Atomic Structures to Low-resolution Electron Density Maps by Surface Overlap Maximization. Journal of Molecular Biology.

[106] Volkmann Niels, Hanein Dorit (1999). Quantitative Fitting of Atomic Models into Observed Densities Derived by Electron Microscopy. Journal of Structural Biology.

[107] Rossmann Michael G., Bernal Ricardo, Pletnev Sergei V. (2001). Combining Electron Microscopic with X-Ray Crystallographic Structures. Journal of Structural Biology.

[108] Chiu Wah, Baker Matthew L, Jiang Wen, Zhou Z.Hong (2002). Deriving folds of macromolecular complexes through electron cryomicroscopy and bioinformatics approaches. Current Opinion in Structural Biology.

